# Supplementary figures and images for: Divergent transcriptional and transforming properties of PAX3-FOXO1 and PAX7-FOXO1 paralogs
Source: PLoS Genet. 2022 May 23;18(5):e1009782. doi: 10.1371/journal.pgen.1009782 (PMC9166405; doi:10.1371/journal.pgen.1009782)

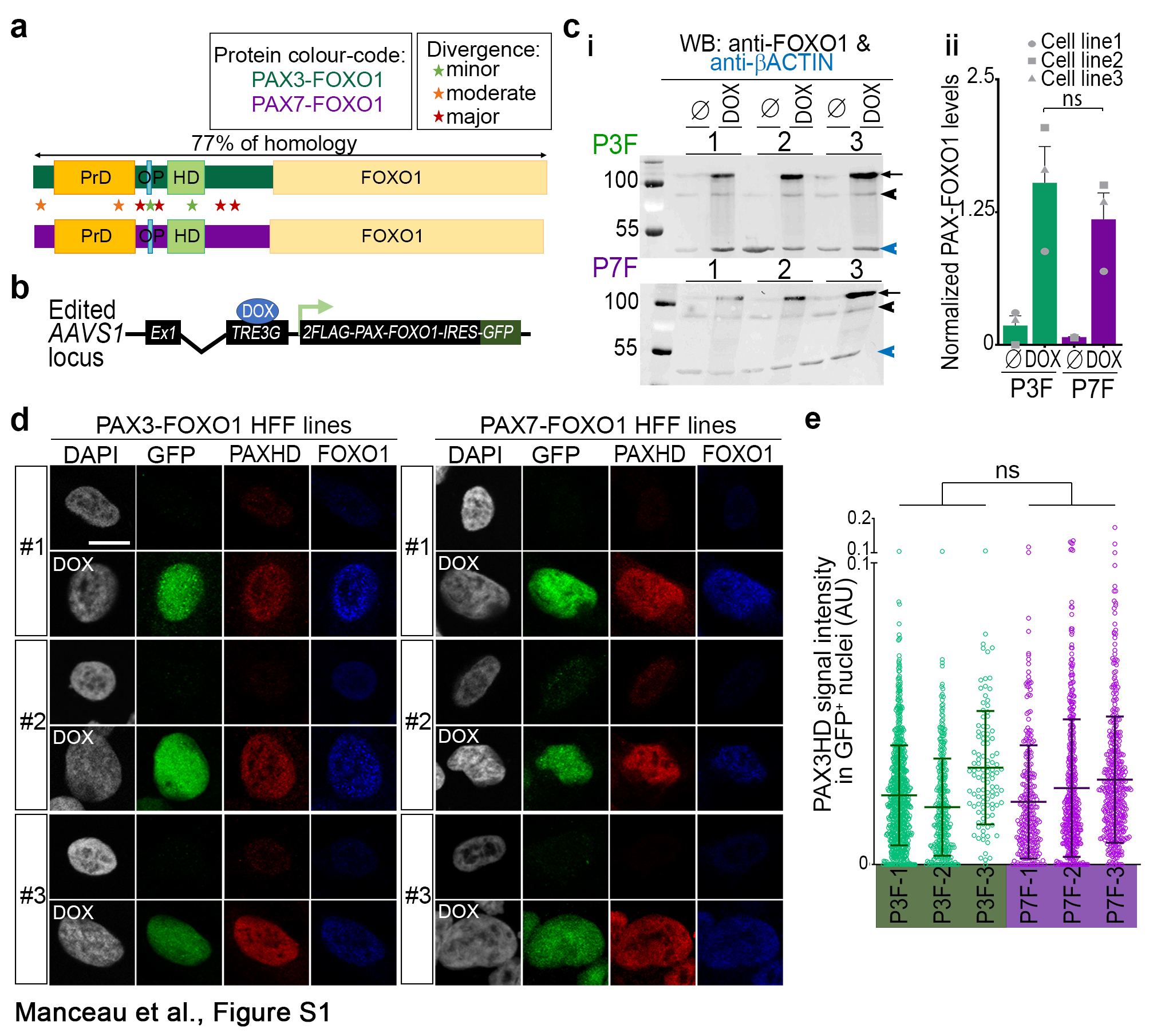

Supplement: S1 Fig — (a) Schematics showing the homogenous and diverging structural protein domains of PAX3-FOXO1 and PAX7-FOXO1. The two proteins share 77% of their amino-acid sequence. The position of the domains displaying divergence in their amino-acid sequence are highlighted by colour coded stars; green stars: <5% of divergence; orange stars: <20% of divergence; red stars: >20% of divergence. HD: homeodomain; OP: octapeptide; PrD: Paired DNA binding domain. (b-e) Levels of expression of PAX3-FOXO1 and PAX7-FOXO1 in DOX inducible HFF lines. (b) Stable HFF lines were generated by introducing a DOX-inducible 2xFLAG-PAX3-FOXO1 or 2xFLAG-PAX7-FOXO1 cassette between exons 1 and 2 of the PPP1R12C gene at the AAVS1 locus. (c)(i) Western blots using FOXO1 and βACTIN antibodies on protein extracts from three independent HFF cell lines (1 to 3) expressing either PAX3-FOXO1 (P3F) or PAX7-FOXO1 (P7F), untreated (Ø) and treated with DOX for 48h (DOX). Arrows indicate PAX-FOXO1 band, black arrowheads point at FOXO1 band and the blue one at βACTIN. (ii) Relative PAX-FOXO1 levels to that of βACTIN quantified from western blots in (i). (d) DAPI staining and immuno-staining using antibodies recognizing GFP, PAX homeodomain (HD) and FOXO1 C-terminal domain on the indicated HFF cell lines treated for 48h with DOX (bottom panels) or without (top panels). Only upon DOX treatment a specific nuclear signal is revealed by the three antibodies in the 3 independent lines expressing PAX3-FOXO1 or PAX7-FOXO1 (#1 to 3). Scale bar: 10μm. (e) Quantification of the levels of PAX-HD signal in GFP+ cells showing an equivalent dispersion of signal levels in PAX3-FOXO1 (P3F) and PAX7-FOXO1 (P7F) expressing cell lines (dots: value for a cell in arbitrary units (AU); bars: mean ± s.d.; ns: ANOVA p-value higher than 0.05). (TIF) [file pgen.1009782.s001.tif]

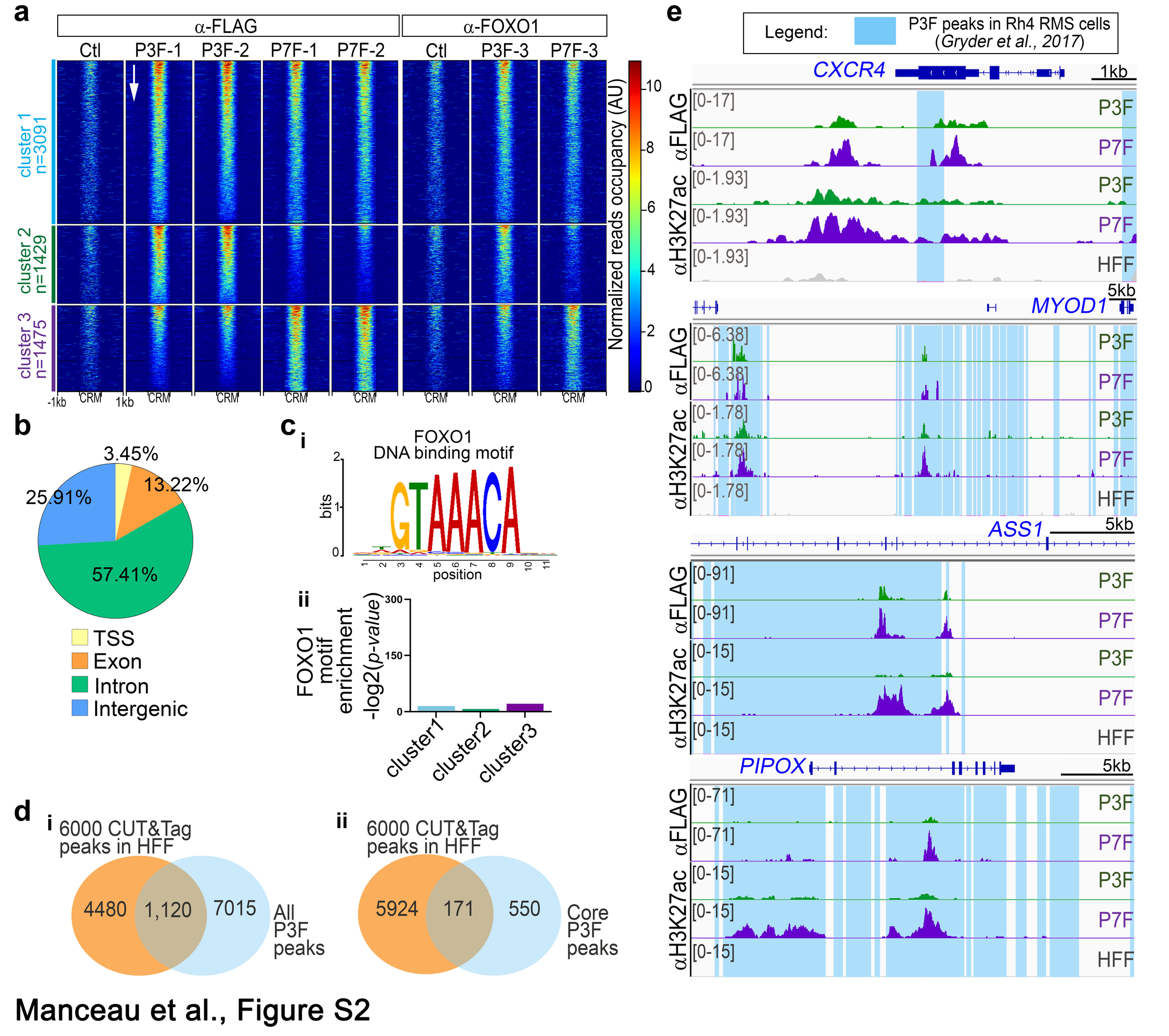

Supplement: S2 Fig — (a) Heatmaps of normalized CUT&Tag signals obtained using either an anti-FLAG or an anti-FOXO1 in control (Ctl) or 3 independent PAX3-FOXO1 (P3F-1 to 3) and PAX7-FOXO1 (P7F-1 to 3) expressing HFF 48h post-DOX treatment in clusters of cis-regulatory modules (CRMs) defined in Fig 1B. Peaks were arranged vertically following the order of signals obtained in P3F-1 sample (cf. arrow). (b) Distribution of the selected 6000 PAX-FOXOs bound CRMs in functional genomic regions (expressed as percentage). TSS: Transcriptional Start Site. (c) i. Logos of position weight matrix of DNA recognition motifs for FOXO1. ii. Enrichment for FOXO1 DNA recognition motif displayed in i in the CRMs belonging to the clusters defined in Fig 1B (bars: -log2(p-value)). (d) Venn diagrams showing the overlap between the 6000 PAX-FOXO1 bound CRMs identified in HFF and all possible PAX3-FOXO1 bound CRMs that have been identified in Rh4, Rh3, SCMC [1] (i) and the overlap between the 6000 PAX-FOXO1 bound CRMs identified in HFF and the list of high confidence PAX3-FOXO1 bound CRMs defined by [1] (ii). (e) IGV tracks showing normalized FLAG and H3K27ac CUT&Tag reads distribution in HFF expressing either PAX3-FOXO1 (P3F) or PAX7-FOXO1 (P7F) 48h post-DOX treatment at four previously identified PAX3-FOXO1 target loci in Rh4 RMS cells [1]. Scales in counts per million (CPM). The peaks of reads overlap greatly with genomic regions identified by ChIPSeq as bound by PAX3-FOXO1 in Rh4, highlighted in blue [1]. (TIF) [file pgen.1009782.s002.tif]

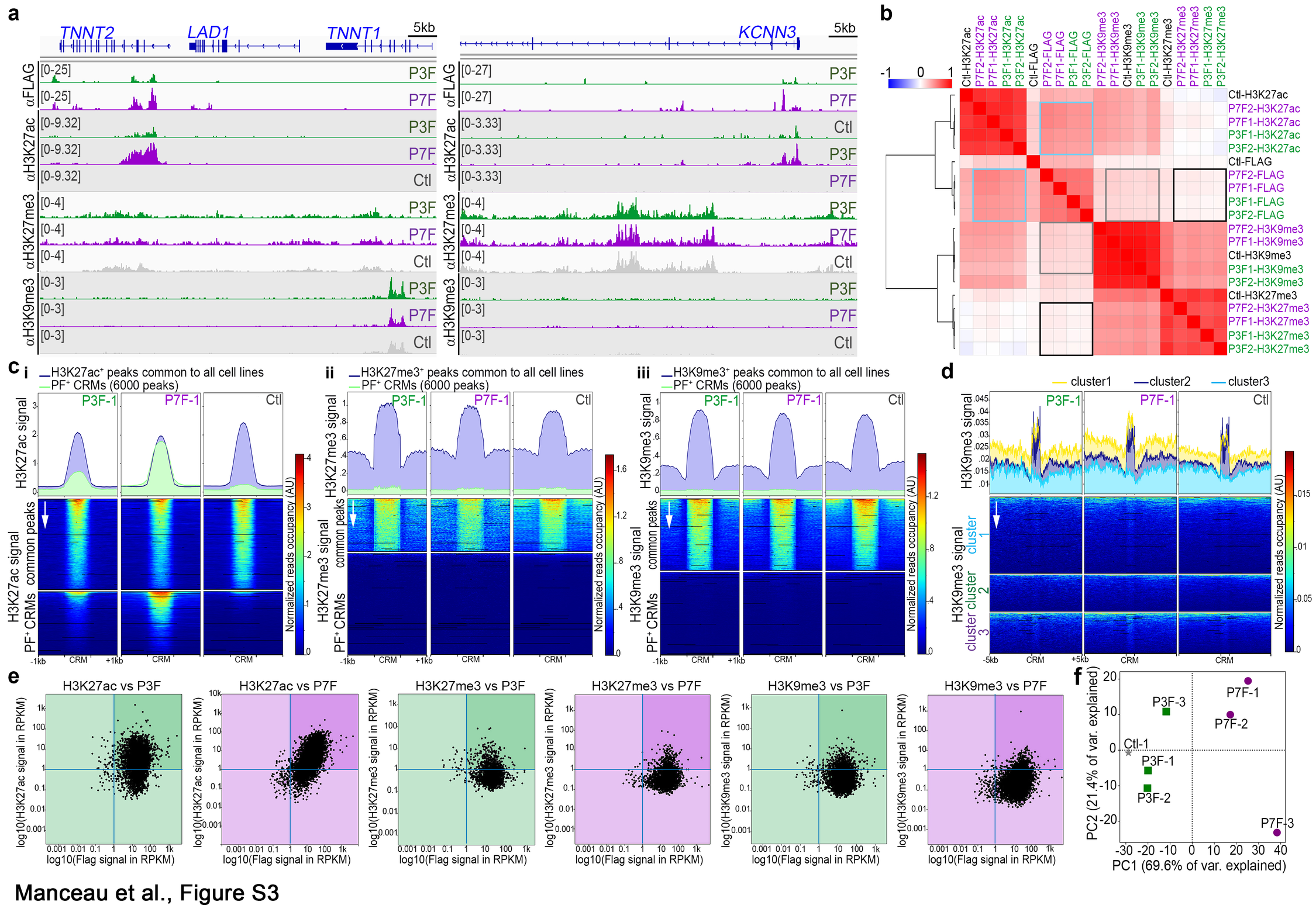

Supplement: S3 Fig — (a) IGV tracks showing normalized FLAG, H3K27ac, H3K27me3 and H3K9me3 CUT&Tag reads distribution in HFF expressing either PAX3-FOXO1 (P3F) or PAX7-FOXO1 (P7F) 48h post-DOX treatment. Scales in counts per million (CPM). They demonstrated the efficiency of the CUT&Tag approach to reveal heterochromatic regions. They also showed at the TNNT2 and KCNN3 loci a poor overlap between PAX-FOXO1 recruitment sites and peak signals for the deposition of the H3K27me3 and H3K9me3 marks. Conversely, they indicated that signals for PAX-FOXO1 binding and H3K27ac deposition coincide. (b) Correlogram displaying the spearman correlations between the genome-wide distributions of FLAG, H3K27ac, H3K27me3 and H3K9me3 CUT&Tag reads in control HFF (Ctl), P3F and P7F cells 48h post-DOX treatment. The correlation between PAX-FOXO1 binding and the H3K27ac deposition signals (blue rectangles) was stronger than that between PAX-FOXO1 binding signals and H3K9me3 or H3K27me3 deposition signals (black rectangles). (c) Average profiles (top panels) and heatmaps (bottom panels) of normalized CUT&Tag signals for the deposition of the indicated histone mark at the 6000 PAX-FOXO1s-bound CRMs defined in Fig 1B (PF+ CRMs) and signal peaks for the indicated histone mark common to all cell lines analysed (common peaks). CUT&Tags were performed in Ctl HFF or expressing P3F and P7F 48h after DOX treatment. The ranking of the CRMs in the heatmaps follows the signal distribution in the P3F sample (cf arrow). Levels of deposition of all three histone marks in common CRMs were comparable between cell lines testifying the robustness in the normalization of CUT&Tag signals. The data presented in both panels also demonstrated that PAX-FOXO1 bound regions were mainly unmarked by the histone modifications, H3K27ac, H3K27me3 and H3K9me3, in control HFF. These regions remained poorly enriched for the deposition of the two heterochromatic histone marks upon PAX-FOXO1s expression. In contrast, a significant number of these [file pgen.1009782.s003.tif]

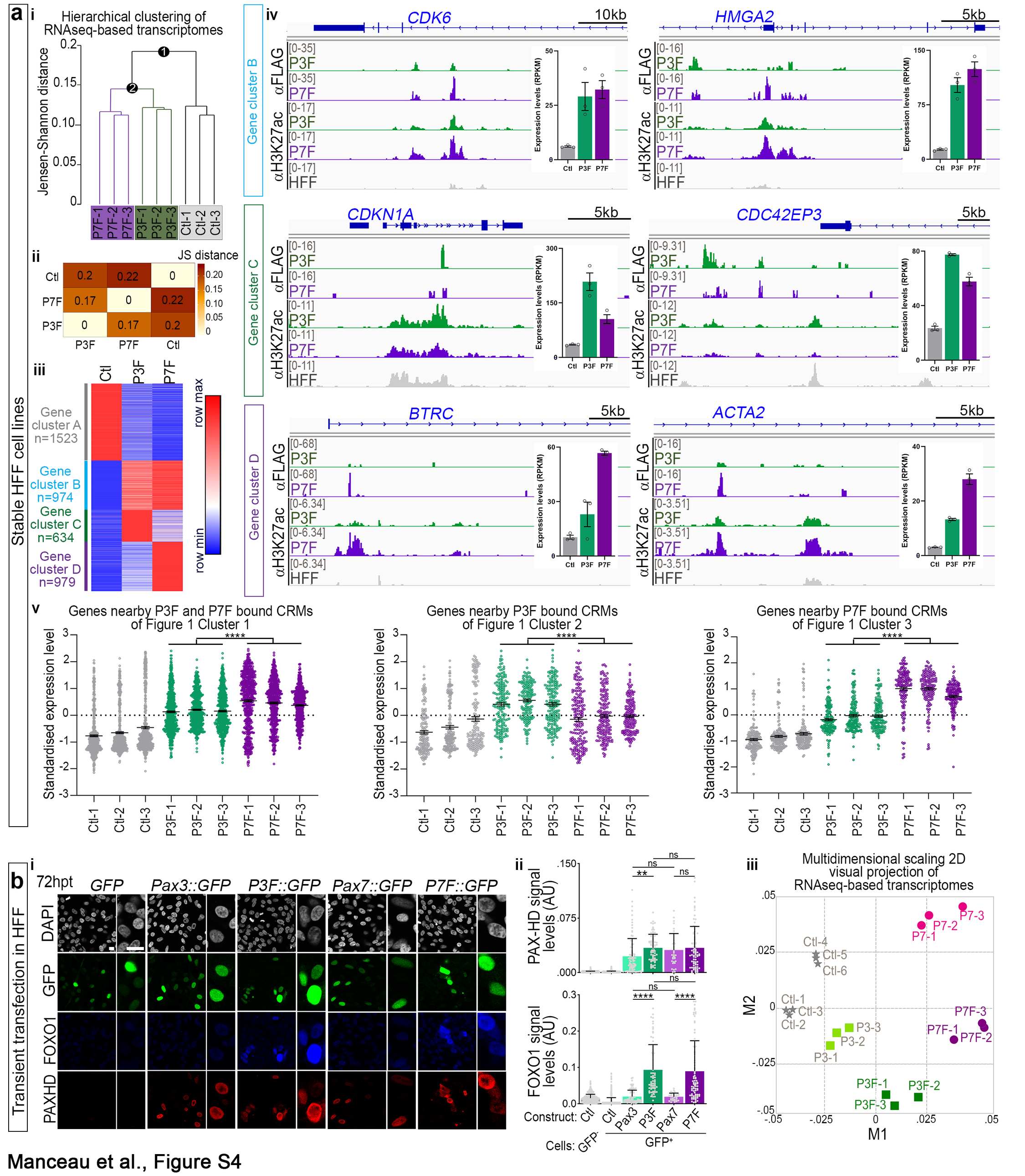

Supplement: S4 Fig — (a) Data obtained on stable HFF lines described in S1 Fig. (ai) Hierarchical clustering based on Jensen-Shannon divergence of RNAseq based transcriptomes of cells described in (Fig 2A) and treated with DOX for 48h. Main segregation nodes have been numbered. The top segregation node separated all PAX-FOXO1 expressing cells from control cells, supporting a primary common mode of action of the two fusion factors. The secondary node clustered all PAX7-FOXO1 (P7F) samples apart from PAX3-FOXO1 (P3F) samples, arguing thus for each PAX-FOXO1 subtype imposing specific transcriptomic traits to cells. (aii) Correlogram displaying the Jensen-Shannon distances between the transcriptomes of control HFF (Ctl), P3F and P7F cells 48h post-DOX treatment. This shows that the transcriptome of P7F cells is more distant from that of Ctl cells than that of P3F cells. (aiii) Heatmap of 4 K-means clusters of genes showing significant variations in their expression between control, PAX3-FOXO1 (P3F) and PAX7-FOXO1 (P7F) expressing cells. Fold changes across samples are colour-coded in blue (minimum levels) to red (maximum levels). (aiv) IGV tracks showing normalized FLAG, H3K27ac, H3K27me3 and H3K9me3 CUT&Tag reads distribution in HFF expressing either PAX3-FOXO1 (P3F) or PAX7-FOXO1 (P7F) 48h post-DOX treatment. Scales in counts per million (CPM). The loci chosen were nearby genes encoding for either cell cycle (left column) or cell shape and motility (right column) regulators and that belong to the gene clusters defined in ii. The bar graphs display the levels of expression assayed by RNAseq for the gene present at these loci in Ctl, P3F and P7F samples. (av) Standardized and normalized expression levels of genes assayed by RNAseq and in the vicinity of CRMs bound by P3F and/or P7F and belonging to the three clusters of CRMs defined in Fig 1B (dots: expression of a given gene; bars: mean±s.e.m.; ANOVA p-value: ****: <0.0001). This indicates that genes nearby CRMs that are bound by P3F and P [file pgen.1009782.s004.tif]

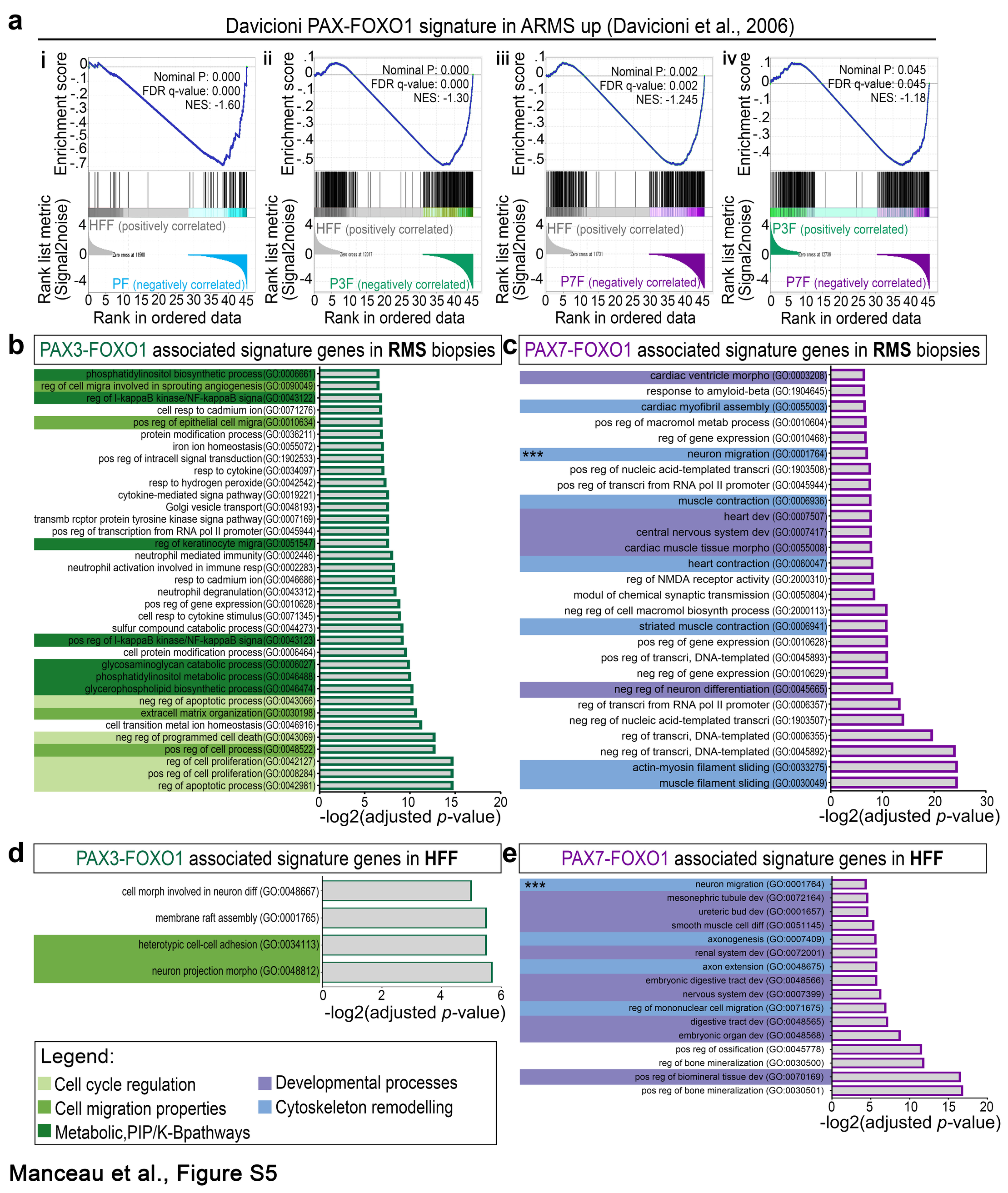

Supplement: S5 Fig — (a) GSEA plots showing the enrichment for genes associated to RMS [2] in differentially expressed genes between control (Ctl) HFF and HFF expressing either PAX-FOXO1s (PF) or PAX3-FOXO1 (P3F) or PAX7-FOXO1 (P7F) or between P3F and P7F samples. NES: normalized enrichment score; FDR: false discovery rate. (b, c) Gene ontology enrichment for biological processes applied to genes enriched in PAX3-FOXO1 RMS biopsies compared to PAX7-FOXO1 FP-RMS biopsies (a) and conversely to genes associated to PAX7-FOXO1 RMS biopsies (b). (d, e) Gene ontology enrichment for biological processes applied to genes enriched in PAX3-FOXO1 expressing HFF compared to PAX7-FOXO1 expressing HFF (c) and conversely to genes associated to PAX7-FOXO1 HFF (d). Related biological processes are highlighted in colour. Bars: -log2(adjusted p-value). (TIF) [file pgen.1009782.s005.tif]

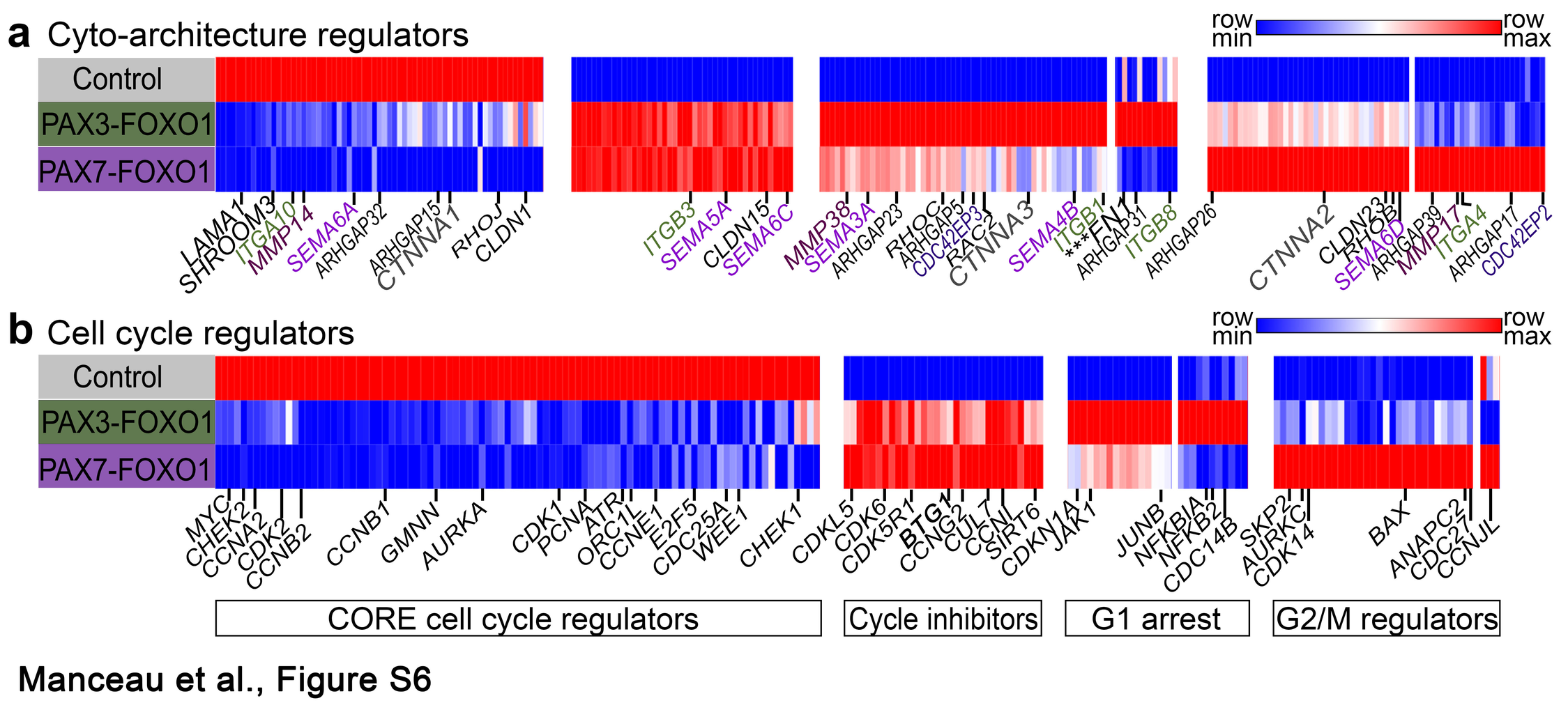

Supplement: S6 Fig — Heatmaps of K-means clustered genes differentially expressed between control HFF, PAX3-FOXO1 and PAX7-FOXO1 stable lines 48h post-DOX treatments. In (a) are clustered genes selected based on the function of the protein they encoded in regulating cell morphology, cell-cell, cell-matrix adhesions, cell motility, while in (b) are grouped genes coding from known cell cycle regulators. In presence of either PAX-FOXO1s, core cell cycle genes, such as checkpoints (CHEK1/2) and Aurora A (AURKA) kinases, the cyclins (CCNA2, B1 and B2) and their dependent kinases (CDK1/2) or the cell-cycle TF MYC are downregulated. Conversely, anti-proliferating genes, including BTG1, CDKL5, CCNG2 or SIRT6 [3–6] are up regulated by both PAX-FOXO1s. PAX7-FOXO1 signature harboured actors of the G2 to M and M to G1 transitions, including members of the anaphase promoting complex (ANAPC2, CDC27) and of the chromosomal passenger complex (AURKC), and modulators of these transitions (CDC14A, SKP2) [7–9]. Conversely, genes more induced by PAX3-FOXO1 were enriched for negative regulators of the G1 to S transition, such as the cyclin inhibitor CDKN1A (p21Cip1) or JUNB [10,11]. Fold changes across P3F and P7F expressing and control HFF are colour coded in blue (lower level) and in red (higher level). Names of genes have been coloured coded as a function of gene families. (TIF) [file pgen.1009782.s006.tif]

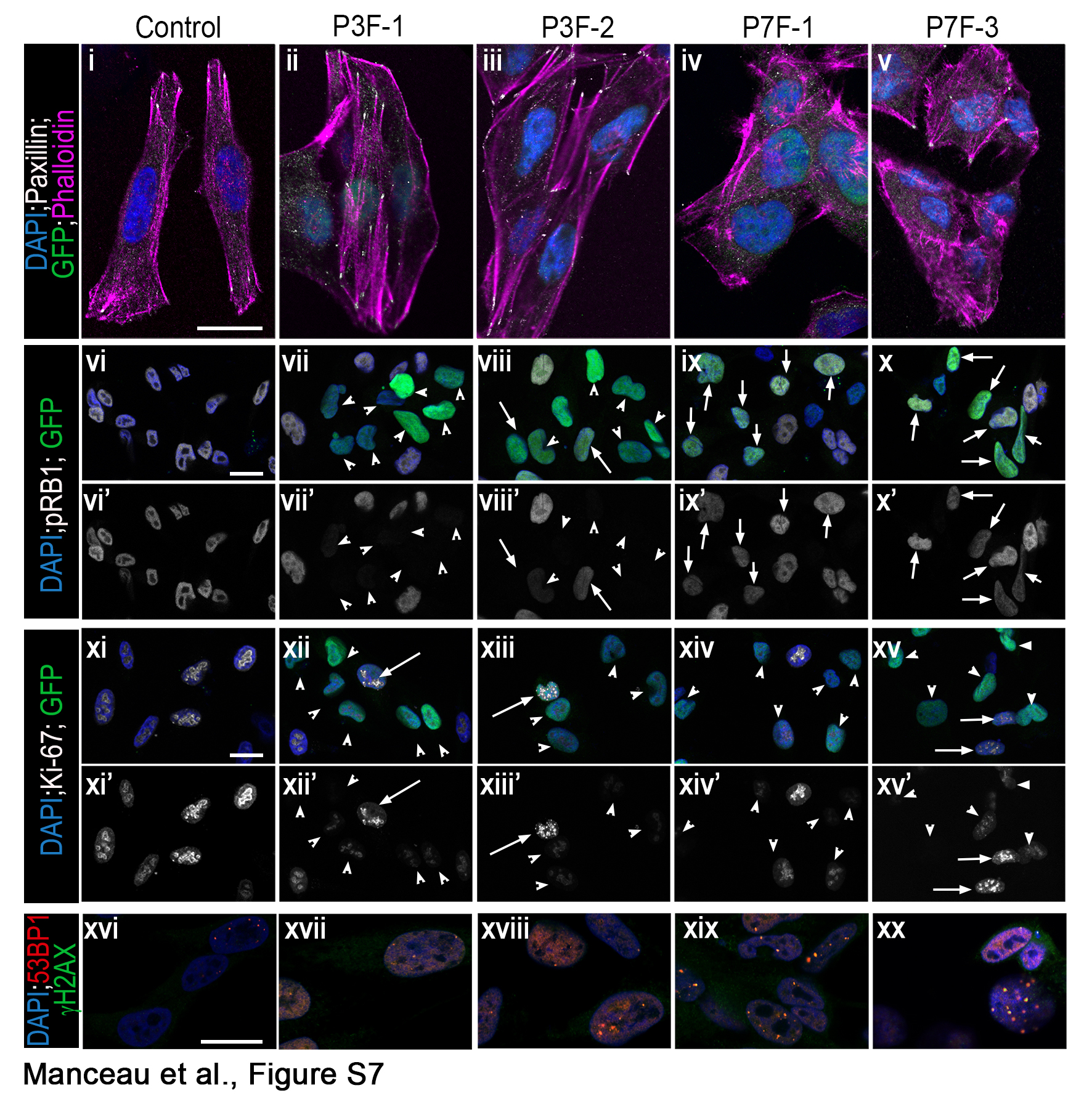

Supplement: S7 Fig — Immunodetection against the indicated proteins, phalloidin staining of F-actin filaments and DAPI labelling in control or two independent PAX3-FOXO1 (P3F-1, 2) and PAX7-FOXO1 (P7F-1, 2) cell lines 48h post-DOX treatment. Arrowheads indicate GFP positive cells showing abnormally low levels of pRB1 or Ki-67 and arrows point at GFP positive cells in which these two proteins can still be detected. All the cells in panel xvii to xx are GFP positive (not shown). Scale bars: 15μm. (TIF) [file pgen.1009782.s007.tif]

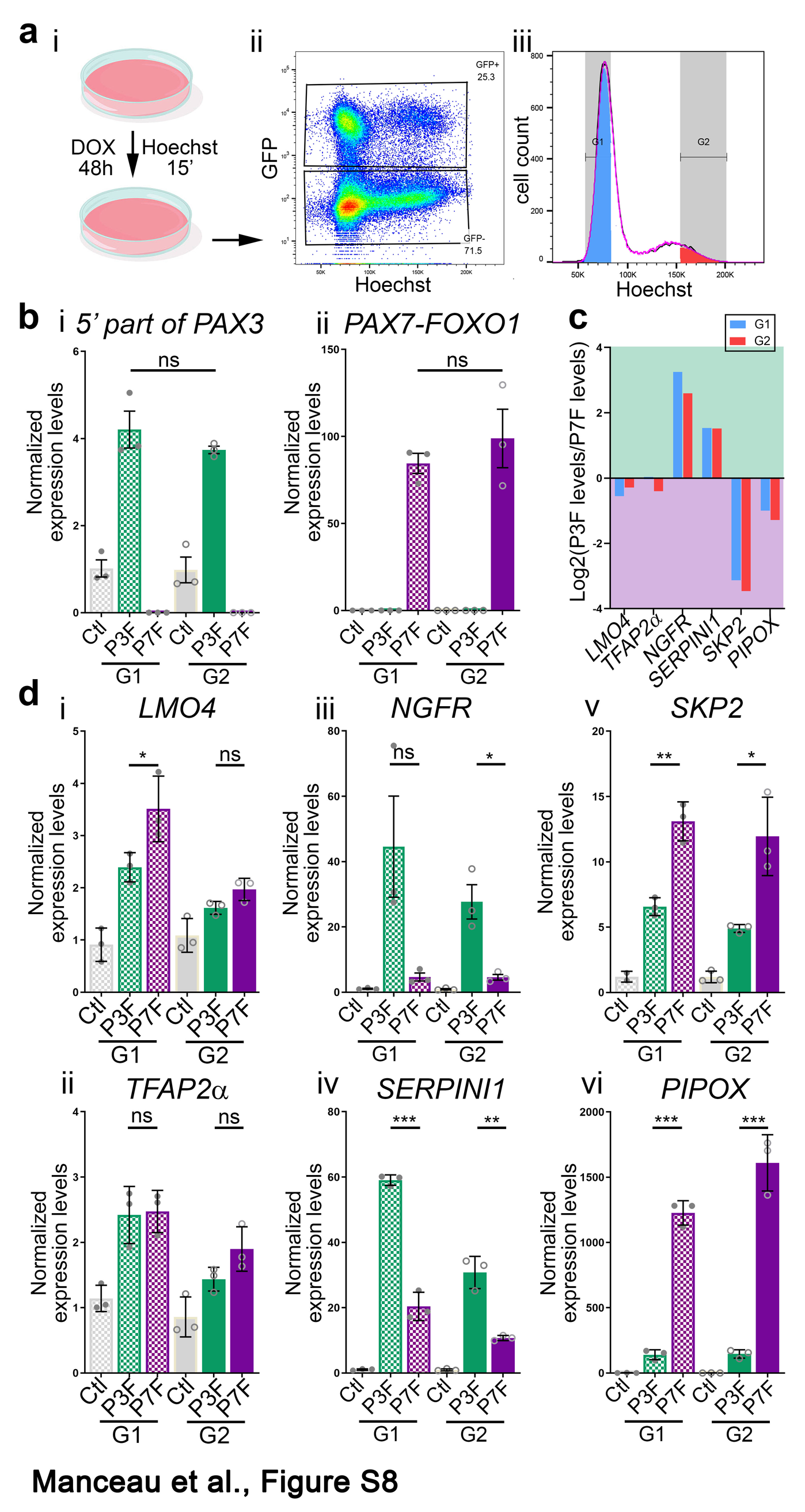

Supplement: S8 Fig — (a) Schematics showing that cells were treated for 48h with DOX and for 15 minutes with Hoechst before preparation for FACS sorts (i). Typical FACS plots illustrating the segregation of GFP+ (PAX-FOXO1+) from GFP- cells (ii; y axis: GFP signal levels; x axis: Hoechst signal intensity) and the selection windows of cells in the G1 (blue) and G2 (pink) phases (iii; y axis: cell count; x axis: Hoechst signal intensity). (b,d) Levels of expression of indicated genes in G1 or G2 phases and in presence or not of PAX3-FOXO1 (P3F) and PAX7-FOXO1 (P7F) (circles: values obtained for independent cell lines, bars: mean ± s.e.m.). This shows that the expression levels of PAX3-FOXO1 and PAX7-FOXO1 induced by DOX from the AAVS1 locus are not submitted to variation throughout the cell cycle (b). For all PAX-FOXO1 targets analysed, their expression levels varied between G1 and G2 phases. In contrast, these targets are in both phases induced by PAX-FOXO1 and their specific sensitivity to PAX3-FOXO1 or PAX7-FOXO1 remains the same. (d). Thus, PAX3-FOXO1 induced NGFR and SERPINI1 to a greater extent than PAX7-FOXO1 regardless of the cycle phase. Similarly, SKP2 and PIPOX showed higher expression levels in the presence of PAX7-FOXO1 than PAX3-FOXO1 in G1 and G2. (c) Graph representing the ratio of the expression levels of the indicated genes in PAX3-FOXO1 cells to those in PAX7-FOXO1 cells in the G1 and G2 phases of the cell cycle, illustrating that the differential response of the cells to a given type of PAX-FOXO1 is not affected by the phase of the cell cycle (bars: log2 of the average over three independent cell lines). (TIF) [file pgen.1009782.s008.tif]
