# Supplementary material for: Divergent transcriptional and transforming properties of PAX3-FOXO1 and PAX7-FOXO1 paralogs
Source: PLoS Genet. 2022 May 23;18(5):e1009782. doi: 10.1371/journal.pgen.1009782 (PMC9166405; doi:10.1371/journal.pgen.1009782)
Supplement: Attachment — Submitted filename: Manceau_et_al_Letter_to_reviewers.pdf [file pgen.1009782.s015.pdf]

We gratefully acknowledge the reviewers for their insightful and encouraging comments. We present here the new data, figures, and writings that address each of the points they raised. All the changes we made in the main and supplementary texts are highlighted in blue. Altogether they have improved and clarified our manuscript and we look forward to referee's thoughts on this revised version of our manuscript.

Reviewer #1:

- minor comments:

(1) "*Both PAX-FOXO1s result in related cell transformation in animal models, but both mutations are associated with distinct pathological manifestations in patients*". Of note, PAX-translocations are not "mutations" but rather alterations. Defining the chromosomal translocations as mutations is incorrect and potentially confusing.

We agree and changed the sentence.

(2) "*the identified pronounced deleterious effects of PAX7-FOXO1 provide an explanation for the low frequency of the translocation generating this factor in patients with rhabdomyosarcoma"* In patients, PAX7 fusions often have better clinical outcomes than PAX3 fusions. The statement, as written might be confusing to readers, suggesting that PAX7-fusions have worse clinical outcomes.

We agree with the referee that this "discussion" sentence at the end of our abstract needed clarification. The ideas behind this proposal cannot be summarized in one sentence, we now develop these ideas only at the end of the paper.

Major comments:

(1) It is of note that PAX3 and PAX7 fusions are expressed as distinct splice forms. This has been known for more than 15 years (PMID: 15688409). In the literature on this topic, PAX7 fusions seem to be co-expressed with about four distinct splice variants, while often the subject of gene amplification events. Merely expressing the fusions in differentiated cells will not recapitulate the clinical/physiological features of these genes as they are expressed in human cancer.

The presence of several PAX3-FOXO1 and PAX7-FOXO1 isoforms with different transcriptional potentials [1] and the specific dynamics of their expression levels in tumours (see for example [2]) are indeed parameters that may modulate the transcriptional response of cells exposed to these fusion factors and that are additional to the modulations presented in our study. This is now acknowledged in discussion sentences. Furthermore, of the splice variants of PAX3-FOXO1 (Q+/Q-) and PAX7-FOXO1 (Q+GL- Q+GL+ Q- GL- Q-GL+) that affect the amino acid sequence of their paired DNA binding domain present in RMS tumours [1], we used the Q+ PAX3-FOXO1 and Q+GL+PAX7-FOXO1 isoforms commonly found in RMS biopsies [1]. The isoforms used in our study are now described in Material and Methods, they consist of two isoforms commonly found in RMS [1].

(2) "*Finally, each of the PAX-FOXO1s could have its own transcriptional activity. Supporting this idea, PAX3-FOXO1 and PAX7-FOXO1 RMS differ in their DNA methylation profiles and transcriptome*". What is the relationship between **chromatin binding and transcription for these fusions**? As written it seems that the authors define **DNA-methylation as a bridge between molecular recognition of DNA and altered transcription**. However, DNA methylation occurs on very slow timescales, while transcriptional activity occurs on very rapid timescales.

We recognize that the first version of our introduction contained an unexplained stretch between the distinct DNA methylation states of PAX3-FOXO1 and PAX7-FOXO1 tumours [3] and the possibility that these two factors harbour distinct transcriptional potential. There is no proof in the literature for a "direct" correlation between PAX-FOXO1 recruitment onto the genome and changes in the methylation of bound CRMs, as previously shown for other transcription factors [4], including the wild-type form of Pax7 [5]. Nonetheless, DNA methylation is a great landmark of the transcriptional state of cells. To wit, promoters and enhancers that display high levels of methylation are generally inactive [6]. In addition, genomic patterns of DNA methylation, including genomic elements relevant for transcriptional regulation such as promoters and enhancers, are dynamically and tightly regulated during cell fate transitions both in development and tumorigenesis [7] and can be modulated at specific regions by TFs [4]. In light of all this, the fact that DNA methylation patterns represent one of the best readouts for

distinguishing PAX3-FOXO1 and PAX7-FOXO1 tumours [3] strongly supports the idea that each fusion protein is likely contributing to direct RMS cells towards a specific transcriptional state.
We have remodelled our sentence in the introduction accordingly.

(3) "*PAX3-FOXO1 binding to non-coding cis-regulatory modules (CRMs) is mediated by its PrD alone or in combination with its HD, but rarely by its HD alone* [15,16]" The conclusion that the FOXO domains do not play a role in chromatin recognition seems to be based on incomplete evidence. What is the evidence that both parts of the fusion do not regulate DNA recognition? The experimental design is potentially problematic, "Human foreskin fibroblasts (HFF) were engineered to express a copy of a FLAG-tagged version of these TFs in a doxycycline (DOX)-inducible manner from the AAVS1 safeguard locus (Figure S1b)." By introducing a tag onto the fusion oncoprotein, the sterics of chromatin recognition will likely be altered. Thus, conclusions from this type of tag-based system are specific for the FLAG-fusions, more so than the endogenous PAX3/7-FOXO1 fusions found in patients.
The enrichment of DNA motifs recognised by the PAX DNA-binding domains indicates the involvement of these protein domains in PAX-FOXO1 DNA recognition, but does not exclude the possibility that the FOXO1 moiety may modulate this recognition. To address this issue, we have
i) been more precise in the formulation of our descriptions.
ii) assessed the enrichment of FOXO1 DNA-binding motifs in the identified PAX-FOXO1 bound CRMs (Figure S2c). As others have previously demonstrated, the enrichment of DNA motifs recognised by FOXO1 is at least 100 times lower than that of motifs recognised by PAXs (Figure S2c) [11–13]. Thus, the specific DNA binding of the fusion proteins is predominantly due to the DNA binding domains of PAX.
iii) further discussed the repertoire of DNA motifs onto which the PAX-FOXO1 can bind to in distinct cellular contexts.

(4) "*Using these cell lines, we first mapped the genomic targets of PAX3-FOXO1 and PAX7- FOXO1 using Cleavage Under Targets and Tagmentation (CUT&Tag) experiments* [22] (Figure 92 1; S2)." The molecular basis of **CUT&Tag** relies on an "input" sample that is essentially ATAC-seq, or open chromatin. Then, the "IP" relies on the pre-condition of chromatin that is already accessible in order to isolate epitope specific DNA-protein binding events. The system is based on selecting accessible DNA-binding sites for the fusion oncoproteins. **Recent evidence in the field suggests that repressive or quiescent chromatin might serve as key targets for these fusions**, and this information cannot be captured with this experimental system.
The CUT&Tag protocol can be used to map the genomic location of proteins associated with both euchromatin and heterochromatin [14,15]. As such, we were able to map the heterochromatin marks, H3K9me3 and H3K27me3 in HFF expressing and not expressing the fusion proteins (Figure S3 and points below). Notably, H3K9me3 was shown to flank PAX3-FOXO1-bound regions in the Rh4 lineage [11]. It was hypothesised that this profile emanates from PAX3-FOXO1 recruitment to heterochromatin. Our CUT&Tags revealed that the CRMs on which PAX-FOXO1 is recruited are initially poorly enriched for these histone marks, nor for H3K27ac. Thus, in HFF, PAX-FOXO1s are mainly recruited to unmarked chromatin. Secondly, the levels of H3K27me3 and H3K9me3 deposition at PAX-FOXO1-bound CRMs and their vicinity (up to 5 kb apart) remain low upon PAX-FOXO1 recruitment and compared to the levels identified at H3K27me3 and H3K9me3 peaks present elsewhere on the genome. Thus, PAX-FOXO1s activity does not interfere with the transcriptional repression mechanisms associated with the deposition of these two marks.

In addition, in light of the reviewer's questions, we decided to modify the representation of PAX-FOXO1 occupancy and H3K27ac deposition rate in the CRMs bound by PAX-FOXO1s (Figure 1b, S2a). In the previous version of the manuscript, the ranking of the CRMs in each heatmap was performed independently of the ranking in the other heatmaps, but was performed according to the amount of signal per CRMs in each sample. From now on, the ranking of CRMs in heatmaps present in a given figure panel is the same and followed that of one sample marked by a white arrow in figures. The ranking is also acknowledged in the figure legend.

(5) Throughout the text, it is suggested that the HD and PD domains (PAX domains) of the fusions regulate DNA binding. However, then it is stated that "*the induction of specific gene cohorts by one of the PAX-FOXO1 chimeras is a property that emanates from its PAX moiety, whereas the induction of common genes is property of the fusion protein*." It is difficult to "rule out" any contributions from the FOXO domain in chromatin recognition of unique sites, to result in distinct transcriptional outcomes. Moreover, it is not discussed in the text whether there are apparent gene-repressive roles for the fusions as well. This should be stated, and elaborated upon. Presenting or focusing only on gene-activating roles is inconsistent with key studies in the field, and potentially driven from the experimental techniques used in this study. This should be included, but the limitations of the CUT& system (see above) might make the argument for gene repression more difficult to describe with the proposed experimental model.
Regarding the role of FOXO1 moiety in the recognition of DNA sites see the answer to point 3. As mentioned in point 4, the chromatin status of PAX-FOXO1 bound regions does not argue for a transcriptional repressive role of these factors. We discuss our results notably in the light of the study by the Stanton's lab [11].

(6) The cell cycle features of the two oncoproteins are meritorious of further mechanistic examination. The **preference for G1/S vs G2/M phases for the PX3 and PAX7-fusions** should be discussed also in the context of previous established studies on "checkpoint adaptation" for PAX3FOXO1 (PMID: 24453992).
To address this point, we have first assessed whether the expression and the transcriptional potential of PAX3-FOXO1 and PAX7-FOXO1 in our inducible HFF lines displayed variation during the course of cell cycle (new Figure S8). Indeed, the study by Kikuchi *et al* [16] showed that PAX3-FOXO1 exhibited high levels of expression and activity in G2/M. The authors were able to show that this dynamic was partly due to the activity of the promoter and regulatory regions in the mouse *Pax3* locus and in the human *PAX3-FOXO1* chimeric locus. Using a DOX-inducible promoter, we observed that this transcriptional regulation across cycle phases does not operate in our HFF cell lines. The expression levels of PAX3-FOXO1 and PAX7-FOXO1 are comparable in G1 and G2. Furthermore, we could show that the differential sensitivity to PAX3-FOXO1 and PAX7-FOXO1 of six genes does not fluctuate according to these phases (Figure S8c,d). These results sustain the idea that the differential transcriptional potential between the two factors shown in our study (Figure 1&2) is not due to the differential modulation of the cell cycle by the two fusion TFs (Figure 3).

Second, we discuss the possible mechanisms by which cells expressing PAX3-FOXO1 and PAX7-FOXO1 could continue their cell cycle and overcome checkpoint cycle arrest that usually follow the appearance of DNA damages.

Altogether, the study is promising, but the methodological limitations especially for the genome-wide binding studies make it challenging to evaluate the conclusions in the context of the approaches and data presented. Connections with cell cycle specific expression (PMID: 24453992), gene amplification for fusions (PMID: 15688409), and sampling chromatin outside of accessible regions would strengthen this study.
All these issues have now been addressed.

Reviewer #2:
(1) The authors show sites of PAX3- and PAX7-FOXO1 binding, and related H3K27ac that tracks along with clusters 1/2/3 that have P3F/P7F. **At these same sites, what is the signal from publically available H3K27ac (cell lines and tumors) or PAX3-FOXO1 signal (in cell lines)**? Note: H3K27ac data from PAX7-FOXO1 primary tumor has been deposited, but not analyzed for the kinds of comparisons done between P7F and P3F in this paper. It would be interesting here if the authors directly compared H3K27ac from a PAX7-FOXO1 tumor (https://www.ncbi.nlm.nih.gov/geo/query/acc.cgi?acc=GSM2214089) to H3K27ac from a PAX3-FOXO1 tumor (https://www.ncbi.nlm.nih.gov/geo/query/acc.cgi?acc=GSM4058221). PAX3-NCOA1 ChIP-seq is in the iScience paper (https://pubmed.ncbi.nlm.nih.gov/32416589/) but also hasn't been analyzed genome wide (https://www.ncbi.nlm.nih.gov/geo/query/acc.cgi?acc=GSM4058219).
We thank the referee for pointing at these data.

Genome-wide comparison of all possible H3K27ac positive peaks that could be found in these PAX3-FOXO1-, PAX3-NCO1A-, or PAX7-FOXO1-positive tumours showed that the H3K27ac deposition profiles of PAX3-FOXO1 and PAX3-NCOA1 tumours were highly comparable, yet diverged from that of PAX7-FOXO1 tumours (Referee Figure 1a). It was not possible to determine whether this divergence was due to differential recruitment of PAX-fusion transcription factors or to the cell of origin and "history" of the tumours, as we lacked data on the sites of recruitment of these factors. To circumvent these lack of recruitment data, we analysed heatmaps showing the deposition of H3K27ac in these tumours samples on CRMs identified as bound by PAX3-FOXO1 in Rh4 cell line [11] (Referee Figure 1b). This showed that while in the presence of PAX3-FOXO1 and PAX3-NCOA1 a footprint is identified in the centre of CRMs, this was not the case in the presence of PAX7-FOXO1, suggesting that PAX7-FOXO1 was not recruited to these sites. We thus haven’t incorporated these data in our paper, as it did not allow us to assess whether the two TFs in the tumour context display differential transactivation potential. Also, after this analysis, the distribution profiles of H3K27ac at a few loci in few patient derived tumour lines appeared to be a weak argument in favour of a differential transcriptional activity between PAX3-FOXO1 and PAX7-FOXO1 (cf. Ex Figure 1d), we also had no data for factor recruitment in these cell lines. We therefore removed this data.
Instead, points 1 and 2 raised by reviewer 2 prompted us to further compare the recruitment regions of PAX-FOXO1 in HFF to those present in PAX3-FOXO1 tumours (see point 2). Furthermore, as mentioned in the response to referee 1 comments, we are now further discussing how the activity of the PAX-FOXO1 in tumours can further be modulated in a tumour context (cf. gene amplification/PAX-FOXO1 isoforms/cell of origin…).

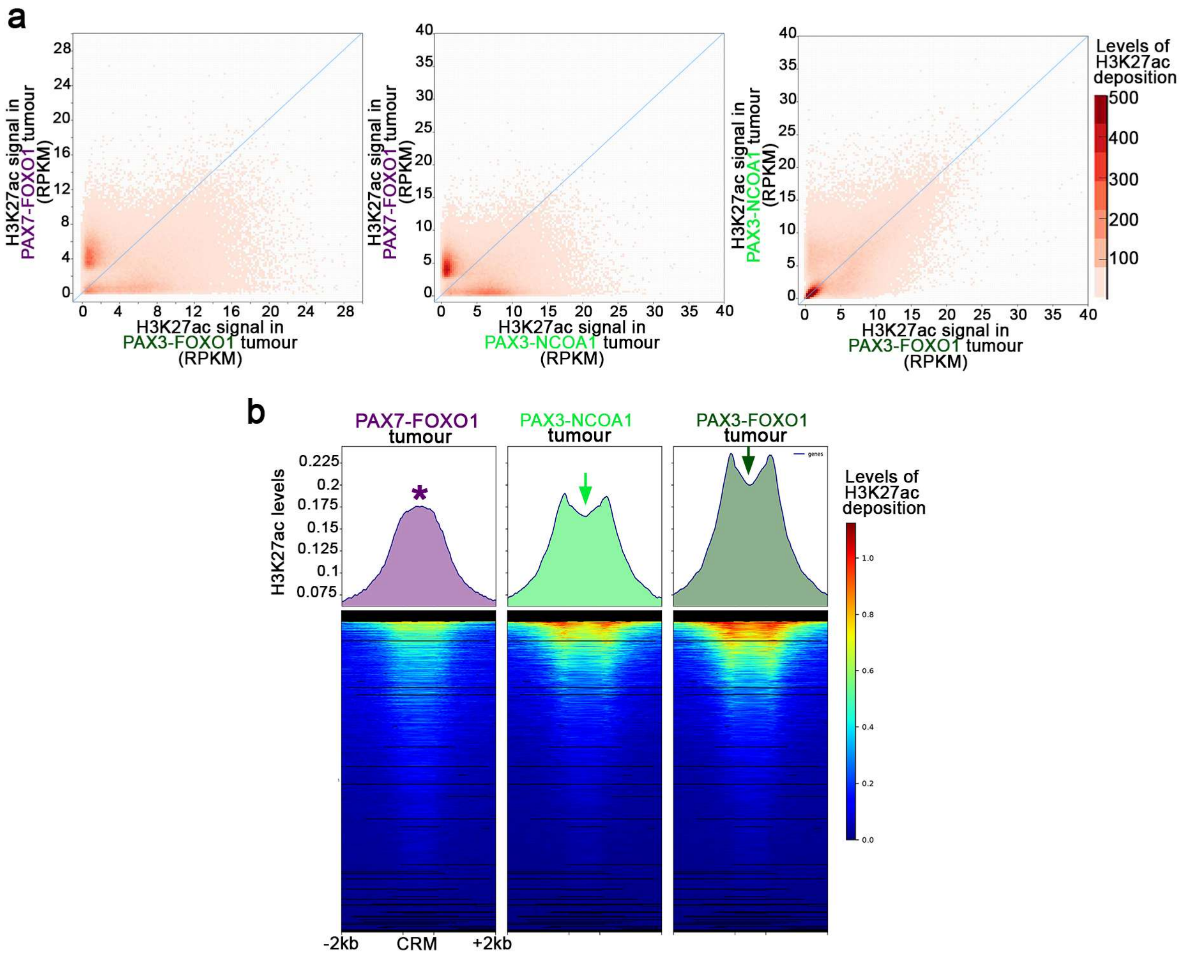

Manceau et al., referee figure 1

Referee figure 1: We first identified all H3K27ac positive CRMs present at least in one of the publicly available ChIPseq data on PAX7-FOXO1 (GSM2214089), PAX3-FOXO1 (GSM4058221) or PAX3-NCOA1 (GSM4058219) tumours. **(a)** Scattered plots showing the H3K27ac signal levels at these CRMs

in one tumour versus another. **(b)** Heatmaps of H3K27ac normalized signal in the indicated tumour sample to the level of CRMs bound by PAX3-FOXO1 in the Rh4 cell line [11].

(2) Two of the most iconic locations of PAX3-FOXO1 binding are at the MYOD1 and FGFR4 super enhancers. Are either of those recapitulated in these CUTandTag experiments?
While PAX3-FOXO1 and PAX7-FOXO1 were found in HFF to be recruited onto two genomic regions 5' to the *MYOD1* genes that are bound by PAX3-FOXO1 in several patients derived cell lines [12,13] (Figure S2d,e), no recruitment of the two factors nearby the *FGFR4* gene was found (not shown). These results pushed us to assess at the genome scale how much PAX-FOXO1 bound CRMs were previously identified as a site of recruitment of PAX3-FOXO1 in tumour cells, including Rh4, Rh3, SCMC [13]. This highlighted that about 18% of the regions we identified in HFFs are known sites of recruitment of PAX3-FOXO1 in tumour cells (Figure S2d). We also decided to show views of the PAX-FOXO1 binding signals in HFF at iconic PAX3-FOXO1 target loci in RMS (Figure S2e). These new data argue for the recruitment of PAX-FOXO1s to the genome being conserved in part in various cellular contexts and across the process of tumorigenesis. Thus, they also support the idea that examining PAX-FOXO1 activity on the fibroblast genome is likely to have a significant impact on the contribution of the two paralogs in establishing the molecular states of the RMS.

(3) Figure 1 panel C would benefit from showing the PrD and HD domains (showing the actual motifs) and using, once, the full name next to the abbreviations PrD and HD.
We followed the reviewer's advice and added the motifs in the main Figure 1 where the full names of the DNA motifs recognized by the DNA-binding domains of PAX-FOXO1 and bHLH TFs are now displayed.

(4) Toward the middle of the manuscript, associated with Figure 2, the authors describe differences in transcriptional output downstream of PAX3-FOXO1/PAX7-FOXO1 activity. In the cell line models, no gene ontology is reported, yet for the primary tumour analysis of public microarray data they do describe sets of gene ontology (ie, cell cycle, cell migration, and metabolism for PAX3-FOXO1 selectively upregulated genes, and embryonic lineage differentiation as well as genes involved in cytoskeletal remodelling for PAX7-FOXO1). The authors need to (a) perform gene ontology from the cell lines models and see if it agrees with their existing analysis, and (b) make two "gene sets" of these 988 and 856 genes (Figure 2d) and use them to perform GSEA on the log2 fold change of expression from their experiments in Figure 2a/c.
Regarding the point a: Gene ontology analysis on the genes which were preferentially induced by PAX3-FOXO1 or PAX7-FOXO1 activity in HFF was already present in our previous version of our manuscript. We have kept this analysis (Figure S5d,e; Table S5).
Regarding the point b: we have performed the proposed analysis (Figure 2eiii, iv).
For interpretation, see point 6.

(5) The paper is all about "Divergent transcriptional and transforming properties of PAX3-FOXO1 and PAX7-FOXO1" and generated new CUTandTag data and new RNA-seq data from a model system in fibroblasts. Based on this, it would be expected that at least 2 types of example gene loci (genome browser shots) would be shown: 1 PAX3-FOXO1 bound and selectively upregulated gene, 1 PAX7-FOXO1 bound and selectively upregulated gene, and 1 gene bound by and upregulated by either P3F/P7F. This kind of "zoom in" to illustrate the transcriptional divergence shown in Figure 2 are warranted.
Such regions are now displayed in Figure S4aiii.

(6) The authors would do well to perform GSEA on the RNA-seq data in cells +/-PAX3-FOXO1 and PAX7-FOXO1, specifically using some of the MolSigDB genesets known for PAX3-FOXO1"
https://www.gsea-msigdb.org/gsea/msigdb/cards/EBAUER_MYOGENIC_TARGETS_OF_PAX3_FOXO1_FUSION
https://www.gsea-msigdb.org/gsea/msigdb/geneset_page.jsp?geneSetName=GRYDER_PAX3FOXO1_ENHANCERS_KO_DOWN
https://www.gsea-msigdb.org/gsea/msigdb/geneset_page.jsp?geneSetName=DAVICIONI_PAX_FOXO1_SIGNATURE_IN_ARMS_UP
We have followed the suggestion of the reviewer; these analyses can now be found in Figure 2e & S5a.

Altogether the GSEA analyses suggested in point 4 and 6 by the reviewer further showed that

i) Genes induced in HFF by PAX3-FOXO1 and/or PAX7-FOXO1 are significantly enriched for genes defining the molecular identity of RMS or previously identified PAX3-FOXO1 targets in RMS. This is consistent with 18% of PAX-FOXO1 bound CRMs identified in HFF were found to be also PAX3-FOXO1 recruitment sites in RMS cells.

ii) PAX7-FOXO1 specific gene signatures in HFF and in RMS are significantly overlapping. This is less pronounced for PAX3-FOXO1 specific gene signatures in HFF and RMS.

(7) "Deeper alterations of cell architecture and cell cycle induced by PAX7-FOXO1 than by PAX3-FOXO1" is missing a verb, such as "are induced". I would recommend active voice here anyway, so "PAX7-FOXO1 induces deeper alterations in cell architecture and cell cycle than PAX3-FOXO1".
Following the reviewer's advice, we have changed all our titles to add a verb.

Reviewer #3:
In their manuscript, Manceau et al. present a deep analysis of the effects of PAX3-FOXO1 and PAX7-FOXO1 on the function of normal human fibroblasts. The goals of the study are clear – to describe the distinct and common functions of these chimeric fusion genes on the transcriptome, epigenome and cell biology of untransformed cells. This is conducted through the generation of models of epitope-tagged PAX3-FOXO1 and PAX7-FOXO1 in HFF cells, followed by study of genome-wide localization of these fusion proteins by CUT&TAG, then analysis of transcriptomics including some fusion with their epigenomic data, and finally, a study of the cell biology of this de novo system. In general, the data is internally consistent, well presented and intriguing. I do, however, have three major concerns and several minor concerns that should be addressed prior to the manuscript being suitable for publication. The goals of my comments are to strengthen this manuscript and ensure experimental rigor.

Major concerns:
(1) In their CUT&TAG analysis, the authors present data to demonstrate that levels of the H3K27ac mark is higher in PAX7-FOXO1 expressing samples, compared with PAX3-FOXO1 expressing samples. In a review of their text, legends and methods section, I am unable to determine if these samples were spiked-in with an exogenous control of some sort. E.coli DNA would be standard for CUT&TAG analysis, and this would be used for normalization of the data. In the absence of this important control, the statement that PAX7-FOXO1 binding is higher than PAX3-FOXO1 binding is difficult to draw and challenging to interpret. Was this control performed? If not, how were the data normalized?
We haven't used an exogenous control DNA in our CUT&Tag assays and have used a normalization taking into account the library size, as recurrently done in ChipSeq, CUT&RUN and CUT&Tag data treatment [17,18]. We have used deeptools to perform this normalization, resulting in Counts Per Million (CPM) measurements. This is now better described in our material and methods.

After our normalization procedure, the coverage of H3K37ac, H3K9me3 and H3K27 deposition assessed at the peak level for these marks commonly found in all cell lines is highly reminiscent in PAX3-FOXO1^{+}, PAX7-FOXO1^{+} and HFF control (Figure S3d). This demonstrates the robustness of our normalization procedure. These observations validate the observed differential in H3K27ac deposition rates at the PAX3-FOXO1 and PAX7-FOXO1 bound CRMs shown in the clusters in Figure 1b.

(2) In general, I find several examples of data that are over-interpreted. The manuscript is of interest by itself, and does not require the data to be "s old." I am listing several examples below, which should be corrected, however, there may be further examples.
To tackle this point, we have more systematically defined the terms we chose and made sure to distinguish what was shown from interpretations and opening discussions. In addition to the points a to c, several writing changes in blue in the text can be found in the new version.
a. Line 127: "*Therefore, both factors induce active chromatin signatures in healthy cells.*" The data here argues that PAX/FOXO fusion proteins are found at sites marked by H3K27ac. Presence of the factor is not the same as chromatin remodeling and establishment of H3K27ac – essentially, induction of the chromatin signature. Recent work by Ben Stanton's group (iScience 2021) seems to suggest PAX3-

FOXO1 is a pioneer factor, which would argue this point, but the data as presented in the paper does not substantiate the claim. Rather, it reflects a correlation. Also, the point of "healthy" cells is unclear. Do the authors mean untransformed cells?
We have rephrased our sentence. In addition, we have further analysed the chromatin status of CRMs onto which the PAX-FOXO1 are recruited in HFFs (see referee 1 point 4). This has allowed us to discuss further our data and compare it to that of the group of Ben Stanton.

b. Line 153: "*Thus, PAX7-FOXO1 also emerges as a more potent transactivator than PAX3-FOXO1 in fully transformed contexts*." This claim is based on the finding that H3K27ac is higher in one PAX7-FOXO1+ cell line (RMZ-RC2), and not in another (CW9019) which has lower levels of PAX7-FOXO1. Most importantly, co-binding is not evidence of gene transactivation, which requires evidence of gene upregulation (not discussed until later in the manuscript). This statement should be softened appropriately.
This data is no longer presented.

c. Line 231: "This would certainly lead to two discrete modes of tissue invasions." This statement immediately follows a segment describing changes in nuclear architecture. I believe the authors have demonstrated intriguing data on actin stress fiber formation. This may be what the authors are referring to, though even actin stress fiber formation is not causal for tissue invasion. If the authors wish to include this statement in the manuscript, it would be important to substantiate that these cells are, in fact, invasive, and that PAX3-FOXO1 and PAX7-FOXO1 positive cells have different methods of tissue invasion. In the absence of this data, I would strongly suggest this statement be removed and the text be clarified.
We have monitored the motility of our cells in live. They barely moved. Movements were mainly due to mitosis. Hence, we could not test properly whether the discrete dynamics in the shape of cells upon PAX3-FOXO1 and PAX7-FOXO1 could change cell motility feature. We have rephrased our discussion.

d. Line 252: "PAX7-FOXO1 was more prone to cause genomic instabilities than PAX3-FOXO1." This statement is over-interpreted. The authors demonstrate increased gamma-H2AX foci, which is a marker of DNA double strand breaks. Is it possible that PAX7-FOXO1 causes a preferential defect of DNA repair mechanisms? Fundamentally, increased double strand breaks are not the same thing as whole genome instability. This statement should be softened/clarified.
We have rephrased our statement

(3). The authors present an intriguing analysis of PAX3-FOXO1 and PAX7-FOXO1 signature genes in Figure S4a. Does this signature correlate to the signature induced in the HFF cells? I can imagine that it may not, due to expression of the transgenes in untransformed cells, though if there are correlations, then this would be interesting to comment on. If there are not, the authors should explain why not in the text.
To address this issue, we have followed the advice of the referee 2 and conducted a series of GSEA analysis showing the significant overlap between the genes whose expression is induced by the PAX-FOXO1 in HFF and RMS gene signatures.
Yet, the overlap is not of 100% of course, we have listed several of the parameters that could modulate PAX-FOXO1 activity, including cell of origin and levels and dynamics of PAX-FOXO1 expression.

Minor Concerns:
1. Many contractions are not defined in the manuscript – notable "CRMs," "PrD", "HD," "bHLH," "TF" and so on. Careful attention to the writing of the manuscript should be paid in order to ensure that abbreviations are defined and the manuscript is clear.
In order to improve the readability of our manuscript and to make the abbreviations accessible we:
(i) verified that they are defined before any use in each section of the text. For example, the abbreviation CRM (cis-regulatory modules) is explained in the introduction, the results & discussion, each figure legend and the supplementary file.

(ii) showed in Figure 1 the DNA motifs present in the PAX-FOXO1-linked regions of the genome and above write the full names of the motifs followed by their abbreviations.

2. As a control in Figure 1d, the authors should present H3K27ac data in normal HFF cells.
The deposition rates of the H3K27ac mark in HFF control cells on CRMs on which PAX-FOXO1 can bind are presented in Figure 1a,b,d, S2c,d and on CRMs that are not bound by PAX-FOXO1 are presented in Figure S2e.

3. The western in Figure S1 appears to demonstrate that FOXO1 expression is increased by doxycycline – is this true/recurrent? Is it due to the transgene or doxycycline? The authors should explain this.
In response to the reviewer, we re-run western blots so that we could not only quantify PAX-FOXO1s and FOXO1 levels using anti-FOXO1 but also β-ACTIN with an antibody directed against this protein (new Figure S1c, Referee Figure 2). By normalizing protein levels by those of β-ACTIN, we show that FOXO1 levels are not induced by PAX-FOXO1 (Referee Figure 2) and that PAX3-FOXO1 and PAX7-FOXO1 levels are comparable (Figure S1c).

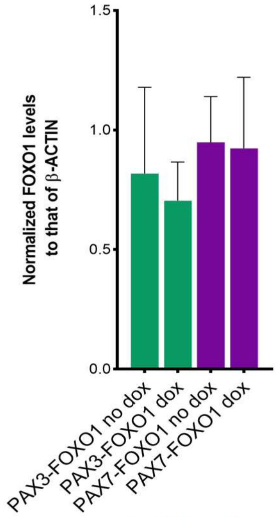

Referee Figure 2: Quantification of FOXO1 protein levels normalized to that of β-actin from the gel displayed in Figure i Sci (means+/-s.e.m) showing that no significant variations in the PAX-FOXO1 inducible HFF lines.

4. H3K27ac is a chromatin mark. The term "occupancy" refers to transcription factors or other proteins that bind to DNA.
We changed the text accordingly.

5. The authors should include a statement that other translocations are found in the disease (i.e. NCOA2, VGLL2).
We have changed the text accordingly and mention these translocations in the final discussion.

6. Some evidence of expression of murine Pax3 or Pax7 should be presented – either qPCR or ideally a western blot – to support Fig 2c. Further, the authors refer only to expression in "cells" – do they mean HFF cells?
We performed immunofluorescence labelling to address this point, to account for intercellular variability. These results are shown in Figure S4b.

7. Are RDabl cells the same as RD?
In the new version of the manuscript we have removed all the data related to RMS cell lines.

8. Are there effects from integrating into the PPP1R12C locus?
To address this, we checked whether few of the parameters we have analysed in the stable line could be reproducible in transient transfected cells (Referee Figure 3).

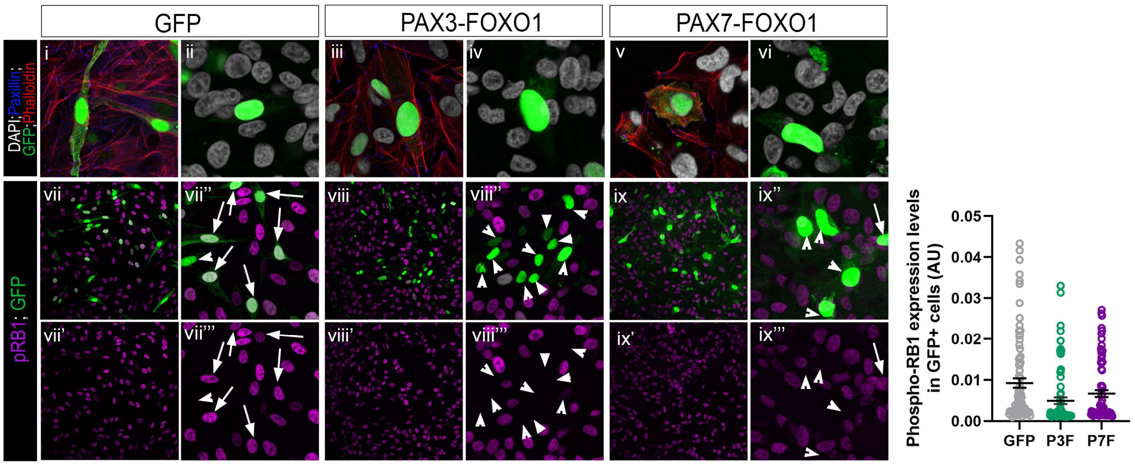

Referee Figure 3: Analysis of few cellular features in HFF 72hours post-transfection with plasmids expressing GFP, PAX3-FOXO1::IRES::GFP or PAX7-FOXO1::IRES::GFP. The pictures show the distribution of the indicated immuno-labelled proteins or Phalloidin marked F-actin and DAPI staining. PAX3-FOXO1+ cells remained elongated as GFP+ HFF (compare iii to i), while the cells adopted a round shape upon expression of PAX7-FOXO1 (v). Nuclei of PAX7-FOXO1 cells appeared to display a reduced sphericity (vi). PhosphoRb1 (pRb1) levels were reduced by the present of PAX-FOXO1 (vii-ix and graph at right). Arrows point at GFP+ and pRb1+ cells, while arrow-heads point at GFP^{+}; $phosphoRb1^{-}$ cells.

References

1. Du S, Lawrence EJ, Strzelecki D, Rajput P, Xia SJ, Gottesman DM, et al. Co-expression of alternatively spliced forms of PAX3, PAX7, PAX3-FKHR and PAX7-FKHR with distinct DNA binding and transactivation properties in rhabdomyosarcoma. Int J Cancer. 2005;115: 85–92. doi:10.1002/ijc.20844

2. Azorsa DO, Bode PK, Wachtel M, Cheuk ATC, Meltzer PS, Vokuhl C, et al. Immunohistochemical detection of PAX-FOXO1 fusion proteins in alveolar rhabdomyosarcoma using breakpoint specific monoclonal antibodies. Mod Pathol. 2021;34: 748–757. doi:10.1038/s41379-020-00719-0

3. Sun W, Chatterjee B, Shern JF, Patidar R, Song Y, Wang Y, et al. Relationship of DNA methylation to mutational changes and transcriptional organization in fusion-positive and fusion-negative rhabdomyosarcoma. Int J Cancer. 2019;144: 2707–2717. doi:10.1002/ijc.32006

4. Lienert F, Wirbelauer C, Som I, Dean A, Mohn F, Schübeler D. Identification of genetic elements that autonomously determine DNA methylation states. Nat Genet. 2011;43: 1091–1097. doi:10.1038/ng.946

5. Pelletier A, Mayran A, Gouhier A, Omichinski JG, Balsalobre A, Drouin J. Pax7 pioneer factor action requires both paired and homeo DNA binding domains. : 13.

6. Greenberg MVC, Bourc'his D. The diverse roles of DNA methylation in mammalian development and disease. Nat Rev Mol Cell Biol. 2019;20: 590–607. doi:10.1038/s41580-019-0159-6

7. Smith ZD, Shi J, Gu H, Donaghey J, Clement K, Cacchiarelli D, et al. Epigenetic restriction of extraembryonic lineages mirrors the somatic transition to cancer. Nature. 2017;549: 543–547. doi:10.1038/nature23891

8. Sun W, Chatterjee B, Shern JF, Patidar R, Song Y, Wang Y, et al. Relationship of DNA methylation to mutational changes and transcriptional organization in fusion-positive and fusion-negative rhabdomyosarcoma. Int J Cancer. 2019;144: 2707–2717. doi:10.1002/ijc.32006

9. Davicioni E, Graf Finckenstein F, Shahbazian V, Buckley JD, Triche TJ, Anderson MJ. Identification of a PAX-FKHR Gene Expression Signature that Defines Molecular Classes and Determines the Prognosis of Alveolar Rhabdomyosarcomas. Cancer Res. 2006;66: 6936–6946. doi:10.1158/0008-5472.CAN-05-4578

10. Laé M, Ahn E, Mercado G, Chuai S, Edgar M, Pawel B, et al. Global gene expression profiling of PAX-FKHR fusion-positive alveolar and PAX-FKHR fusion-negative embryonal rhabdomyosarcomas. J Pathol. 2007;212: 143–151. doi:10.1002/path.2170

11. Sunkel BD, Wang M, LaHaye S, Kelly BJ, Fitch JR, Barr FG, et al. Evidence of pioneer factor activity of an oncogenic fusion transcription factor. iScience. 2021;24: 102867. doi:10.1016/j.isci.2021.102867

12. Cao L, Yu Y, Bilke S, Walker RL, Mayeenuddin LH, Azorsa DO, et al. Genome-Wide Identification of PAX3-FKHR Binding Sites in Rhabdomyosarcoma Reveals Candidate Target Genes Important for Development and Cancer. Cancer Res. 2010;70: 6497–6508. doi:10.1158/0008-5472.CAN-10-0582

13. Gryder BE, Yohe ME, Chou H-C, Zhang X, Marques J, Wachtel M, et al. PAX3–FOXO1 Establishes Myogenic Super Enhancers and Confers BET Bromodomain Vulnerability. Cancer Discov. 2017;7: 884–899. doi:10.1158/2159-8290.CD-16-1297

14. Kaya-Okur HS, Janssens DH, Henikoff JG, Ahmad K, Henikoff S. Efficient low-cost chromatin profiling with CUT&Tag. Nat Protoc. 2020;15: 3264–3283. doi:10.1038/s41596-020-0373-x

15. Ahmad K, Henikoff S. The H3.3K27M oncohistone antagonizes reprogramming in Drosophila. Bosco G, editor. PLoS Genet. 2021;17: e1009225. doi:10.1371/journal.pgen.1009225

16. Kikuchi K, Hettmer S, Aslam MI, Michalek JE, Laub W, Wilky BA, et al. Cell-Cycle Dependent Expression of a Translocation-Mediated Fusion Oncogene Mediates Checkpoint Adaptation in Rhabdomyosarcoma. Horwitz MS, editor. PLoS Genet. 2014;10: e1004107. doi:10.1371/journal.pgen.1004107

17. Meers MP, Tenenbaum D, Henikoff S. Peak calling by Sparse Enrichment Analysis for CUT&RUN chromatin profiling. Epigenetics & Chromatin. 2019;12: 42. doi:10.1186/s13072-019-0287-4

18. Zhu Q, Liu N, Orkin SH, Yuan G-C. CUT&RUNTools: a flexible pipeline for CUT&RUN processing and footprint analysis. Genome Biol. 2019;20: 192. doi:10.1186/s13059-019-1802-4
