# Supplementary material for: Divergent transcriptional and transforming properties of PAX3-FOXO1 and PAX7-FOXO1 paralogs
Source: PLoS Genet. 2022 May 23;18(5):e1009782. doi: 10.1371/journal.pgen.1009782 (PMC9166405; doi:10.1371/journal.pgen.1009782)
Supplement: Attachment — Submitted filename: Manceau_et_al_Referees_Revision2_Final.pdf [file pgen.1009782.s016.pdf]

We thank the referees for their positive and constructive comments. We detail below the elements (figures and text) that we have modified to improve the clarity of our manuscript and in accordance with all their advice. In addition, we have made minor changes (such as typos) in the course of our revisions, they also appear in blue in the text.

Reviewer #2:

The authors have nicely addressed all my concerns, and the paper is improved greatly. I'm happy to recommend publication.

We thank the referee for his support.

Reviewer #3:

Manceau et al present a revised manuscript, defining the effects of PAX3-FOXO1 and PAX7-FOXO1 on the transcriptome and epigenome of fibroblasts. It is clear they have done much work in this revision, and the manuscript is improved as a result. Despite this, I still find issues that must be addressed for this manuscript to be acceptable for publication. I do not think further experiments need to be done - rather, I think that the authors need to carefully edit this manuscript for clarity and to ensure their statements reflect their findings.

1. The authors broadly overstate their findings in several places. The most concerning of these overstatements is found in the abstract, summary, results and discussion, where the authors claim that PAX7-FOXO1 has "higher transactivation potential" than PAX3-FOXO1. Their data as presented does not support this very broad claim. At no point do the authors demonstrate that the **whole transcriptomic effects** of PAX7-FOXO1 expression are greater than those of PAX3-FOXO1. This could be achieved by a violin plot of all expressed genes in these different scenarios.

To complete the hierarchical clustering of the transcriptomes of PAX3-FOXO1 and PAX7-FOXO1 cells shown in Figure S4ai, we now provide a correlogram showing that the transcriptome of PAX7-FOXO1 cells diverged the most from PAX3-FOXO1 cells and control HFF (Figure S4aii).

Alternatively, **the authors could focus on genes bound by both PAX3-FOXO1 and PAX7-FOXO1 and examine the actual gene expression values in their RNAseq data**. Is the expression higher for PAX7-FOXO1 than PAX3-FOXO1 at loci bound by both these proteins? The authors attempt an analysis like this in Fig 2C, however, this is a row-normalized heatmap, and a scatterplot or bar plot with an appropriate statistical test would actually prove this.

We now provide plots of the expression levels of genes nearby the CRMs bound by PAX3-FOXO1 and/or PAX7-FOXO1 (Figure S4av). This notably indicates that genes nearby CRMs onto which both PAX3-FOXO1 and PAX7-FOXO1 can be recruited are induced in presence of both PAX-FOXO1s but displayed higher expression levels in PAX7-FOXO1 cells than in PAX3-FOXO1 cells. Furthermore, while the genes in the vicinity of CRMs bound by one of the PAX-FOXO1s show higher expression levels in cells expressing that PAX-FOXO1, the induction levels of genes nearby PAX7-FOXO1-specific CRMs are much higher than that of genes nearby PAX3-FOXO1-specific CRMs. This further sustains the idea that PAX7-FOXO1 displays a greater transactivation potential than PAX3-FOXO1, overall at the genome level.

Regardless, this point is actually not exceptionally important. PAX3-FOXO1 and PAX7-FOXO1 bind to similar and distinct regions of the genome, both function as pioneer factors, and cause distinct functional outcomes. Claims of higher or lower transactivation, in the absence of showing actual gene transactivation, are really unnecessary. To this end, I strongly recommend the authors simply remove this concept of "higher" or "lower" transactivation from

their manuscript. Their data does not support this statement, and it doesn't really add much to their paper.
We now provide further evidence for our previous conclusions. Yet, we have been more precise in our abstract and the description of Figure 1, in order to avoid any overstatement.

Other examples of broad overstatements:
Line 168-175: Here, the authors describe that H3K27ac is deposited at CRMs higher at P7F loci than P3F loci. The authors go on to discuss that this means that there is higher transactivation potential for P7F. H3K27ac is a histone mark, associated with promoters and enhancers. It correlates with expressed genes, but is also found at poised loci which are not expressed. It is an overreach to claim that more H3k27ac means more gene transactivation, without providing further evidence.
The data presented above and the remodelling of the text we have made shall answer to the reviewer. Yet, we may say that we do not agree with the referee's remark.
First, PAX-FOXO1's binding mainly take place at distal *cis*-regulatory elements of the genome and not at promoters, so all the deposition of H3K27ac that is described in our manuscript are mainly taken place onto enhancers and not promoters [1] (Figure S2b).
Second, the deposition of H3K27ac described in the paper is not at poised enhancers. Indeed, if such poised enhancers are defined by features of active enhancers, such as the presence of transcription factors, co-activators (e.g. p300) and the deposition of H3K4me1, yet they are poorly marked by H3K27ac[2]. Furthermore, the repressive mark H3K27me3 is usually found at poised enhancers, which, as we have shown, is not the case in PAX-FOXO1-bound sites (Figure S3). Finally, the transcriptomic profiles of genes nearby CRMs that are bound by both PAX7-FOXO1 and PAX3-FOXO1 argues that the levels of deposition of H3K27ac to these CRMs correlate largely with the levels of expression of these genes (Figure S4av).

Line 302-304: Here, the authors describe that they will test whether PAX-FOXO cell cycle deregulation is linked to DNA damage. They then go on to show that DNA ds breaks are elevated in PAX7-FOXO1 expressing cells, compared with PAX3-FOXO1, and then claim that these means more genome instability. This section needs to be re-written. The authors have shown correlative data to suggest that PAX7-FOXO1 expression is associated with increased DNA ds breaks, and have not linked this to cell cycle control at all. Further, the presence of DNA ds breaks is not the same thing as genome instability.
We have taken into account this remark and modified the corresponding paragraph in the result section.

2. Minor text edits:
Line 123-124: The authors state that they identified 6000 CRMs with PAX3-FOXO1 and/or PAX7-FOXO1 binding signals. In the methods, however, they state that they took the "top 6000" binding signals. Which is true?
We have clarified this in the results section to match methods section.

Comments on RMS cell lines should be taken out of the text, now that they are not used in this manuscript anymore.
This was done by remodelling the acknowledgement section.

The discussion of EWS-FLI and drawing parallels to PAX3-FOXO1 and PAX7-FOXO1 in the discussion is confusing.
We have removed it.

Reviewer #4:

Manceau et al directly compares the genome binding of PAX3-FOXO1 and PAX7-FOXO1, associated histone marks, and transcriptional consequences in the context of human fibroblasts. This has been a longstanding question in the rhabdomyosarcoma field, why PAX7-FOXO1 has different clinical outcomes than PAX3-FOXO1 positive tumors. This is a challenging question to address given the lack of animal models and high toxicity of the fusion-oncogenes in cell culture systems. This study represents a first step to understand PAX3/7-FOXO1 divergent activities and ties their genomic data to phenotypic consequences detailed in Figure 3 related to cellular morphology, cell cycle arrest, and DNA integrity. The authors have addressed the reviewer comments satisfactorily and have presented new data that has improved the overall study. Additional clarification on whether these fibroblasts have the capacity to transform would help frame this study and claims in the context of the field (see below).

Major Comments

1. Additional explanation on the rationale for choosing fibroblasts to express the PAX3/7-FOXO1 fusions. Elaborate on why fibroblasts were chosen for this study instead of myoblasts. Do these PAX3/7-FOXO1 expressing fibroblasts have the capacity to transform, and if not, how would you interpret these findings as being related to the disease? This could be included as part of the discussion.

Several cell types are likely at the origin of RMS, as testified by the tissues in which the primary tumour growths are found. Amongst them, stand the myoblasts and cells of the conjunctive tissues, including mesenchymal stem cells and fibroblasts. All these cell types have previously shown to be responsive to PAX3-FOXO1 and/or PAX7-FOXO1 (e.g.[1,3,4]). This is the primary reason for why we have chosen this cell type. Also, the existing studies on the mode of action of PAX3-FOXO1 in fibroblasts gave us sufficient hindsight to establish a reliable comparison between its mode of action and that of PAX7-FOXO1[1].

We now mention in the discussion that the transcriptional impact of PAX-FOXO1 is like other factors likely to vary with the cell type in which they are expressed. It will be important to analyse this impact in other cell types that may give rise to RMS.

Minor Comments

1) There is no limit to PLOS Genetics manuscript length and the methods can be included in the main text.

We followed the referee's advice.

2) Figure 2A legend does not describe the presented data.

Thank you for pointing this out to us, we have modified it accordingly.

3) Figure 3A is a red/green overlay and should be modified so it is accessible to colour-blind individuals.

Red was replaced by pink in Figure 3A and the related Figure S7.

References

1. Gryder BE, Yohe ME, Chou H-C, Zhang X, Marques J, Wachtel M, et al. PAX3–FOXO1 Establishes Myogenic Super Enhancers and Confers BET Bromodomain Vulnerability. Cancer Discov. 2017;7: 884–899. doi:10.1158/2159-8290.CD-16-1297

2. Creyghton MP, Cheng AW, Welstead GG, Kooistra T, Carey BW, Steine EJ, et al. Histone H3K27ac separates active from poised enhancers and predicts developmental state. Proceedings of the National Academy of Sciences. 2010;107: 21931–21936. doi:10.1073/pnas.1016071107

3. Kendall GC, Watson S, Xu L, LaVigne CA, Murchison W, Rakheja D, et al. PAX3-FOXO1 transgenic zebrafish models identify HES3 as a mediator of rhabdomyosarcoma tumorigenesis. eLife. 2018;7: e33800. doi:10.7554/eLife.33800

4. Pandey PR, Chatterjee B, Olanich ME, Khan J, Miettinen MM, Hewitt SM, et al. PAX3-FOXO1 is essential for tumour initiation and maintenance but not recurrence in a human myoblast model of rhabdomyosarcoma: PAX3-FOXO1 in rhabdomyosarcoma tumourigenesis. J Pathol. 2017;241: 626–637. doi:10.1002/path.4867
